# Cerebellar Contributions to Social Cognition in ASD: A Predictive Processing Framework

**DOI:** 10.3389/fnint.2022.810425

**Published:** 2022-01-28

**Authors:** Isabelle R. Frosch, Vijay A. Mittal, Anila M. D’Mello

**Affiliations:** ^1^Department of Psychology, Northwestern University, Evanston, IL, United States; ^2^Institute for Innovations in Developmental Sciences, Northwestern University, Evanston and Chicago, IL, United States; ^3^Department of Psychiatry, Northwestern University, Chicago, IL, United States; ^4^Department of Medical Social Sciences, Northwestern University, Chicago, IL, United States; ^5^Institute for Policy Research, Northwestern University, Chicago, IL, United States; ^6^McGovern Institute for Brain Research, Massachusetts Institute of Technology, Cambridge, MA, United States

**Keywords:** autism spectrum disorder (ASD), cerebellum, adaptive prediction, predictive processing, neuroimaging, social cognition, language, action perception

## Abstract

Functional, structural, and cytoarchitectural differences in the cerebellum are consistently reported in Autism Spectrum Disorders (ASD). Despite this, the mechanisms governing cerebellar contributions to ASD, particularly within the sociocognitive domain, are not well understood. Recently, it has been suggested that several core features of ASD may be associated with challenges creating and using prior expectations or predictions to rapidly adapt to changing stimuli or situations, also known as adaptive prediction. Importantly, neuroimaging, clinical, and animal work find that the cerebellum supports adaptive prediction in both motor and non-motor domains. Perturbations to the cerebellum *via* injury or neuromodulation have been associated with impairments in predictive skills. Here, we review evidence for a cerebellar role in social cognition and adaptive prediction across individuals with and without ASD.

## Introduction

Differences in social cognition, including interpreting socio-communicative intent from gestures and adapting behaviors to different social contexts are characteristic of Autism Spectrum Disorders (ASD; American Psychiatric Association, 2013). Although many brain regions support social cognition, over the past decades, there has been a growing recognition of the sociocognitive role of the cerebellum in both typical and atypical development (Fatemi et al., 2012; D’Mello and Stoodley, 2015; Stoodley and Tsai, 2021). The importance of the cerebellum to social cognition is particularly evidenced by its involvement in this capacity in ASD: the cerebellum is the most consistently implicated structure in ASD and neuroimaging, clinical, and preclinical studies in ASD consistently report associations between the cerebellum and social behaviors (Steadman et al., 2014, D’Mello and Stoodley, 2015; Ellegood et al., 2015). Moreover, neuromodulation of the cerebellum in ASD mouse models can ameliorate social symptoms (Stoodley et al., 2017). What remains lacking are mechanistic frameworks designed to integrate this wealth of empirical and conceptual work. In the motor realm, the cerebellum is well established as a core structure in adaptive prediction—or the process by which we make and update models of our world to optimize behavior (Ito, 2006). More recently, the field has begun to understand that the cerebellum also contributes to adaptive prediction in social cognition, which requires us to interpret the actions of others, anticipate what they might say and when they might say it, and infer mental states from their actions and words (Koster-Hale and Saxe, 2013; Stoodley and Tsai, 2021). Here, we review the existing evidence for a cerebellar role in adaptive prediction and explore whether differences in cerebellar adaptive prediction may contribute to both strengths and challenges in social cognition in ASD. This review begins by discussing the importance of adaptive prediction for social cognition and then moves to a discussion of the basic organization of the cerebellum as well as empirical evidence for cerebellar contributions to adaptive prediction in social cognition. Next, it turns to examining the literature on differences in adaptive prediction for social cognition in autism, specifically focusing on cerebellar findings. We conclude with directions for future research on cerebellar adaptive prediction and ASD.

## Linking Adaptive Prediction and Social Cognition in ASD

Adaptive prediction facilitates the integration of proximal and distal experiences (Friston, 2005) to render social information processing efficient in the moment. This involves using past experiences to: (1) derive intent from the actions of others; (2) anticipate what they may say; and (3) infer their mental states (i.e., theory of mind or mentalizing) to enable rapid online correction of our own behaviors in response (Koster-Hale and Saxe, 2013). Consider a conversational partner who repeatedly clears their throat. We may infer that they want to interject and respond by pausing. If they do not interject, or if they mention recovering from a cold, we adapt our future behavior accordingly (e.g., pause less or speak louder in response to this behavior). Updating socio-cognitive models with novel information thereby impacts thoughts and actions.

A consistent observation is that some autistic individuals^1^ do not rely on past information to flexibly adjust their behavior and adapt to changing situations (Cannon et al., 2021). This observation has shaped predictive coding theoretical frameworks of ASD and may explain challenges (Pellicano and Burr, 2012; Lawson et al., 2014; Sinha et al., 2014; Van de Cruys et al., 2014) and strengths characteristics of ASD (Rozenkrantz et al., 2021). Autistic self-reports also describe social cognition as an explicit process. In relaying her experience of social cognition to Oliver Sacks, Temple Grandin—a prominent autistic scientist—describes that she had not accumulated the implicit knowledge of social conventions that many non-autistic individuals build up over a lifetime (Sacks, 1993). Rather, her understanding of the intentions, actions, and mental states of others was a logical, computed process, based largely on explicit recall of former experiences and overt associations. She refers to these former experiences as “videos” in her internal “library of experiences”, and describes explicitly coupling these “videos” with extensive research to predict what someone in a certain context might think or do.

## Adaptive Prediction in The Cerebellum: From Neurons to Networks

**Figure 1:**
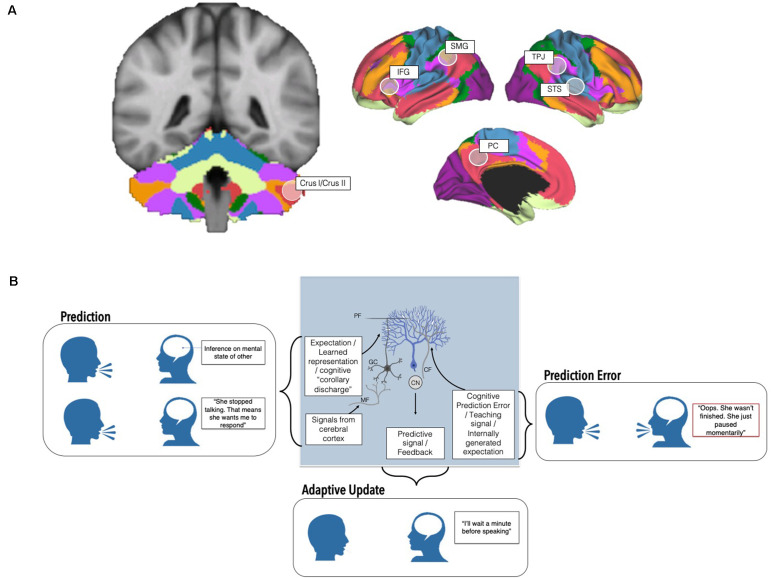
Cerebellar contributions to adaptive prediction in social cognition from neurons to networks. **(A)** The posterolateral cerebellum, particularly Crus I/II, is a node of whole-brain cognitive resting state networks including the Default Mode (red) and Frontoparietal networks (orange; Buckner et al., 2011; Yeo et al., 2011; other networks visualized include dorsal (green) and ventral attention (violet), somatomotor (blue), visual (purple), and limbic (cream)). In addition to resting state networks, several task-based neuroimaging studies find that discrete regions of the cerebellum are maximally engaged by specific tasks (e.g., Stoodley and Schmahmann, 2009; Guell et al., 2018; King et al., 2019). This cerebellar region is also functionally connected with and consistently activated alongside regions implicated in theory of mind (right temporoparietal junction, TPJ, right superior temporal sulcus, STS, and the precuneus, PC) and language processing (left inferior frontal gyrus, IFG, left supramarginal gyrus, SMG). **(B)** At the cellular level, Granule cells (GC) receive input from the rest of the brain and spinal cord *via* mossy fibers (MF) and may transmit expectation-related information *via* their parallel fiber (PF) axons to Purkinje cells, the principal neurons of the cerebellum. On the other hand, prediction errors are carried by climbing fibers (CF) originating in the inferior olive, which also synapse onto Purkinje cells (blue). Climbing fiber input to Purkinje cells is thought to signal which granule cell signals are most important in a given context (Wagner and Luo, 2020 for review). Predictive signals and feedback are ultimately relayed to the rest of the brain *via* output from the cerebellar nuclei (CN). These circuits, and interactions between the cerebellum and the rest of the brain, ultimately enable the creation and deployment of predictions, prediction errors, and adaptive changes to behavior. Altered cerebellar cytoarchitecture, circuitry, and connections with the cerebrum may affect different aspects of adaptive predictions with relevance for sociocognitive challenges in autism.

The cerebellum contains over 50% of the neurons in the central nervous system and plays an important role in modulating motor and cognitive functions. Specific cerebellar subregions are linked to discrete supratentorial regions *via* a series of reciprocal, closed-loop circuits. Cerebellar outputs reach the cortex *via* the thalamus, and input to the cerebellum arrives from the cortex *via* the pons. These loops provide the putative circuitry by which the cerebellum can modulate cortical processes, and also underpin regional specificity in cerebellar topography. For instance, the anterior cerebellum is reciprocally connected to sensorimotor cerebral cortices and is involved in motor behaviors, while the posterolateral cerebellum is connected to non-motor association cortices and is involved in cognitive behaviors (Buckner et al., 2011; Bernard et al., 2012; Figure 1A). Importantly, our understanding of cerebellar functional topography is evolving, and newer studies have shown that the cerebellum houses representations of many discrete cognitive behaviors (D’Mello et al., 2020a) and tasks (King et al., 2019). Unlike the cerebral cortex, cytoarchitecture is consistent throughout the cerebellum. Therefore, it is suggested that the cerebellum conducts one fundamental operation on any input it receives (Schmahmann, 2004; Diedrichsen et al., 2019). In the motor realm, one hypothesis is that this operation involves adaptive prediction, and the underlying mechanics have been studied in detail. Traditional models of cerebellar adaptive prediction hold that copies of motor commands from the motor cortex (“efference copy”) are used to create predictions of the sensory consequences of actions, enabling the cerebellum to optimize actions without needing to wait for sensory feedback which is, by definition, delayed. The cerebellum can adjust these predictions based on sensory feedback. Mismatches between actual and predicted sensory feedback result in sensory prediction errors and updates to the original prediction. Cerebellar predictions and internal models can be refined over time, allowing for rapid, online adaptation of motor behaviors and long-term corrections (Ito, 2006; Shadmehr et al., 2010). At the cellular level, this process is supported in part by granule cells that carry contextual information from the rest of the brain (for example, efference copies, or other learned representations), and synapse onto Purkinje cells (the sole output of the cerebellar cortex) *via* parallel fibers. In addition, climbing fibers from the inferior olive provide prediction error signals to Purkinje cells, which distinguish which granule cell inputs are most informative. These prediction errors can be signed or unsigned, signaling exactly *how* cerebellar Purkinje cells should respond to alter behavior (see Corlett et al., 2022 for evidence of prediction error in human cerebellum). Inputs from climbing fibers can cause long-term depression (LTD) at parallel fiber-Purkinje cell synapses—potentially serving to inhibit actions that resulted in sensory prediction errors (Wagner and Luo, 2020 for review; Figure 1B). Notably, the circuitry underlying cerebellar sensorimotor adaptive prediction is complex, and our understanding of the contributions of specific neuronal subtypes is rapidly evolving (Hull, 2020). For example, there is evidence that cerebellar neurons both scaffold learning how to perform an action, and also learning which action is the correct one to perform in a given context (Medina, 2019). In addition, teaching signals hypothesized to be carried by climbing fibers can be modulated by experience, suggesting that the cerebellum may play a role beyond simple error-based learning (see Hull, 2020 for discussion).

This emerging evidence, coupled with the strikingly uniform cytoarchitecture within the cerebellum, has been used to propose that similar adaptive predictive computations are performed in the posterior cerebellum on sociolinguistic inputs from non-motor regions (Sokolov et al., 2017; though see Diedrichsen et al., 2019 for a discussion on how uniform circuits may not be directly related to uniform function, and evidence for multiple functionalities in the cerebellum). These inputs, coupled with unique regional specialization of cell types, electrophysiological properties, and expression patterns of Purkinje cells in the posterior lobe, likely support more complex sociolinguistic adaptive prediction (Kozareva et al., 2021). In this view, instead of motor efference copies, the posterior cerebellum uses “cognitive” efference copies to form sociocognitive predictions which allow us to anticipate what a social partner is likely to think or say (Van Overwalle et al., 2014). These efference copies may include inferences of another’s mental states arising from theory of mind regions or semantic information from language-relevant regions (Figure 1A). Supporting this, several neuroimaging studies find that the posterolateral cerebellum, particularly lobules VI-VIII, represents sociolinguistic predictions and prediction errors (Moberget and Ivry, 2016; Ernst et al., 2019; Van Overwalle et al., 2019; Corlett et al., 2022). Neuromodulation of these regions perturbs predictive language behaviors and social sequencing (Lesage et al., 2012, 2017; D’Mello et al., 2017; Van Overwalle et al., 2020). Strikingly, cognitive predictions and even violations of cognitive predictions, not just motor predictions (i.e., reward prediction error, reward expectation, reward delivery) can be carried by cerebellar granule cells (Figure 1B). For example, an elegant study in mice found that the cerebellum is monosynaptically connected to the ventral tegmental area (VTA), a key region in reward processing, and that cerebellar neurons that projected to the VTA were preferentially activated when mice engaged in social approach behaviors (Carta et al., 2019). These findings have expanded traditional views of cerebellar circuitry, to incorporate non-motor signals that could influence cognitive behaviors (Wagner and Luo, 2020).

## Cerebellar Adaptive Prediction and Social Cognition: Relevance to Autism Spectrum Disorders

There is a large literature characterizing cerebellar contributions to autism (see Fatemi et al., 2012; D’Mello and Stoodley, 2015 for review). For instance, the most consistent findings in neuroimaging studies of ASD include reduced volume in cerebellar Crus I and II. These regions are thought to be particularly implicated in ASD given their connections with contralateral cerebral sociolinguistic regions, as well as with the default mode and frontoparietal networks (Figure 1A). Postmortem studies in autistic individuals find that Purkinje cell reductions are greatest in Crus I/II (Fatemi et al., 2002; Skefos et al., 2014), and modulation of these regions in animal models of ASD can both cause and rescue social challenges as well as other behaviors characteristic of ASD (Tsai et al., 2012; Stoodley et al., 2017; Badura et al., 2018). Lesions to the posterolateral cerebellum in premature infants, children, and even in adulthood can result in mutism, expressive and receptive language difficulty, and sociocognitive and executive function challenges (e.g., Schmahmann and Sherman, 1998; Limperopoulos et al., 2007; Gudrunardottir et al., 2011; and many others) . Despite existing literatures on the cerebellum and adaptive prediction, the compelling links between the cerebellum and autism, and the interest in adaptive prediction frameworks in autism, few theoretical or empirical studies have explicitly linked these fields. We bridge existing literature across these domains to describe how cerebellar adaptive prediction may contribute to cognitive strengths and challenges in ASD.

### Interpreting the Actions of Others

Human actions are relatively standard, enabling us to form expectations about what movements someone might make next, and use these to understand what goals they are trying to achieve. Action perception—inferring goals from action, and understanding why actions are being performed—relies on sensory inputs, but is largely a cognitive process and scaffolds higher-order inferences necessary for theory of mind. Several empirical studies report that autistic individuals show difficulty inferring goals from others’ actions (Zalla et al., 2010; Schuwerk et al., 2016), using contextual priors to facilitate action prediction (Amoruso et al., 2019) and internally representing the observed actions of others (Cattaneo et al., 2007).

In non-autistic individuals, these inferences engage lobules VI, Crus I/II (Sokolov et al., 2012, 2014; Abdelgabar et al., 2019; Van Overwalle et al., 2020). For example, when shown point-light displays or moving shapes, non-autistics quickly infer biological motion, or even emotionality and intent (Jack and Pelphrey, 2015, Jack et al., 2017). Stronger Crus I/II activation, and increased connectivity between these regions and the posterior superior temporal sulcus (pSTS)—a region implicated in action inference—is associated with increased likelihood of describing motion in social-affective, vs. motor terms. Activation in lobule VI and Crus I/II also reflect imitation and mirroring (inferring the goals of another’s actions by matching them to representations of our own actions; Jack et al., 2011; Van Overwalle et al., 2014). Damage, degeneration, and disruption of the cerebellum can affect action perception (Sokolov et al., 2010). For example, transcranial magnetic stimulation (TMS) to the posterior cerebellum impairs action perception, and the ability to distinguish biological motion from random motion (Ferrari et al., 2019). In addition, cerebellar degeneration in spinocerebellar ataxias is associated with worse action perception ability (Abdelgabar et al., 2019). Children with cerebellar tumors show difficulty using others’ actions to predict and infer outcomes and, unlike typically-developing peers and peers with supratentorial tumors, show no contextual facilitation of action interpretation (Butti et al., 2020). This suggests that cerebellar disruption may uniquely affect the use of contextual priors to predict likely action outcomes.

Few studies have explicitly examined cerebellar contributions to action perception and inference in ASD. One study found reduced activation in the posterior cerebellum of autistic individuals (bilateral Crus I) during action imitation compared to non-autistic participants (Jack and Morris, 2014). Reduced activation in Crus I/II in ASD during biological motion processing was associated with greater parent-reported social challenges (Jack et al., 2017). Moreover, reduced connectivity between right Crus I/II and the contralateral pSTS was associated with parent-reported mentalizing skills in autistic children (Jack and Morris, 2014). Interestingly, atypical connection patterns within this circuit (e.g., increased connectivity in ipsilateral, non-canonical Crus I/II-pSTS circuits) were also associated with self-reported social difficulties (Jack et al., 2017).

### Anticipating the Words of Others

Language comprehension is fundamentally predictive, and prior linguistic knowledge and context help to resolve ambiguity when linguistic input is noisy. Several studies have reported that predictive linguistic processing is altered in ASD and that autistic individuals rely less on context to resolve ambiguous words (though see Hahn et al., 2015). For example, when reading ambiguous words aloud (e.g., “read”), autistic children were less likely to vary their pronunciation as a function of changes in surrounding words (Hala et al., 2007; Wagley et al., 2020). Some studies find that autistic children also do not show neural signatures of faciliatory language processing (e.g., reduced engagement of language regions over several repetitions of surprising linguistic input; fewer changes in linguistic brain regions with increased exposure to speech; Scott-Van Zeeland et al., 2010; Wagley et al., 2020).

Neuroimaging and clinical evidence support a role for the posterior cerebellum in linguistic prediction (see Argyropoulos, 2016 for review). Crus I/II activation increases during the formation of a semantic prediction (Moberget et al., 2014; D’Mello et al., 2017; Lesage et al., 2017), and engagement of these lobules is highest when decisions about semantic plausibility must be made quickly (D’Mello et al., 2020b). Cerebellar activation also represents violations of linguistic predictions, and subsequent adjustments to internal models (Sheu and Desmond, 2021). One study found that improvements in the perception of previously ambiguous words were associated with increased right Crus I activation (Guediche et al., 2014). Cerebellar neuromodulation and damage alter word and phrase-level priming (Argyropoulos, 2011; Gilligan and Rafal, 2019), verb generation, predictive sentence processing, and internal monitoring of speech errors (Gebhart et al., 2002; Stoodley and Schmahmann, 2009; Runnqvist et al., 2016).

Cerebellar contributions to language prediction in ASD have not been directly investigated. However, many studies find reduced cerebellar activation during language processing and decreased connectivity between the cerebellum and cortical language networks in autistic children and adults compared to non-autistic individuals (Verly et al., 2014; D’Mello and Stoodley, 2015). Further, structural and functional differences in the posterior cerebellum have been associated with early language delay and ability in ASD (Verly et al., 2014; D’Mello et al., 2016; Hegarty et al., 2018).

### Inferring the Mental States of Others

Theory of Mind (TOM), or mentalizing, refers to the ability to infer the beliefs, thoughts, and goals of others. This process engages regions across the cerebral cortex including prefrontal areas, the temporo-parietal junction, and the precuneus (see Adolphs, 2009 for review of the TOM network). TOM is perhaps one of the most studied aspects of ASD. Studies find that autistic individuals showed reduced reliance on prior expectations when attempting to infer the intentions of others and that these reductions were associated with higher self-reported social difficulties (Chambon et al., 2017).

Mentalizing reliably engages the posterior cerebellum in non-autistic individuals (Van Overwalle et al., 2014; Nguyen et al., 2017). The cerebellum contains representations of the DMN, a network that overlaps with the TOM network, and contains a fine-grained representation of the temporoparietal junction (TPJ)—a core node of the TOM network (Igelström et al., 2017). Cerebellar damage can result in difficulty with mentalizing, interpreting the emotions of others, and even interpreting social scenes (Van Overwalle et al., 2020; Clausi et al., 2021a). As in the case of predictive language processing, few studies have explicitly linked the cerebellum to mentalizing abilities in ASD and those that do often focus on biological motion and action perception (see section above). However, the putative substrates for cerebellar contributions to mentalizing challenges in ASD exist (Leggio and Olivito, 2018). For instance, autistic individuals show similar TOM profiles to cerebellar lesion patients (e.g., lower scores on Reading the Mind in the Eyes and Faux Pas Tasks) and have overlapping reductions in gray matter (though this study did not find associations between cerebellar volume and TOM scores in either group; Clausi et al., 2021b). Additionally, reduced connectivity between the cerebellum and regions implicated in TOM have been reported in ASD (Khan et al., 2015). One such study reported that while the functional organization of the temporo-parietal junction (TPJ) was intact, there was reduced connectivity between the TPJ and Crus I/II of the cerebellum in autistic individuals (Igelström et al., 2017).

### Altered Cerebellar Adaptive Prediction and Strengths in ASD

The majority of studies examining social cognition, the cerebellum, and adaptive prediction abilities in ASD focus on deficits or challenges in these domains. However, reduced reliance on predictions and past experience can be a strength in ASD, for example in the domains of reasoning, decision making, and cognitive biases (see Rozenkrantz et al., 2021). Despite this, few studies have taken strengths-based approaches when assessing how cerebellar contributions to adaptive prediction may play a role in the heterogeneous profile of strengths and challenges in ASD. One such study demonstrated that Purkinje-cell specific mouse models of ASD (e.g., L7-Tsc1) show *both* sensory deficits and sensory learning strengths (i.e., outperform wildtype mice on a sensory-accumulation learning task; Oostland et al., 2021). One interpretation of this is that reduced predictive capacity as a result of cerebellar impairment can result in an increased focus on recent or incoming sensory information and allow for stronger learning.

## Discussion

Adaptive prediction frameworks have been useful in organizing our understanding of social cognition in ASD and developing hypotheses for future research. The cerebellum, a structure known for its role in adaptive prediction, is often excluded from empirical neuroimaging studies and theoretical discussions of neural substrates of social cognition in ASD. This renders disentangling the lack of cerebellar findings from the lack of cerebellar involvement in these domains difficult. Future research should take a whole-brain approach and strive to interpret cerebellar results within the context of the existing literature when possible. Moreover, some studies report that autistic individuals do not show difficulty using past experience to adapt behavior at lower levels of processing (e.g., visual, motion perception; Sandhu et al., 2019), and that these differences only emerge for higher-order stimuli or demands. Future research should look across levels of processing to determine which aspects of ASD may be best explained by cerebellar-specific adaptive prediction mechanisms. Future studies should also take care to assess whether cerebellar contributions to adaptive prediction can explain the heterogeneous profile of strengths and challenges in ASD. Notably, early injury to the cerebellum can result in the development of ASD-relevant behaviors which persist into adulthood (Wang et al., 2014). Understanding how cerebellar adaptive prediction contributes to socio-cognitive development is especially relevant for ASD—a neurodevelopmental disorder. Lastly, cerebellar differences and atypical adaptive prediction are found in multiple psychiatric and neurodevelopmental disorders. This suggests that the cerebellum might be a domain-general predictive processor across cognitive domains and categorical diagnoses (Sokolov et al., 2010; Diedrichsen et al., 2019; D’Mello and Rozenkrantz, 2020). A transdiagnostic approach to adaptive prediction differences may reveal shared neurobiological mechanisms across neurodevelopmental and psychiatric conditions.

## Author Contributions

IF and AD conceptualized and wrote the initial draft. VM provided feedback and contributed to the final draft. All authors contributed to the article and approved the submitted version.

## Conflict of Interest

The authors declare that the research was conducted in the absence of any commercial or financial relationships that could be construed as a potential conflict of interest.

## Publisher’s Note

All claims expressed in this article are solely those of the authors and do not necessarily represent those of their affiliated organizations, or those of the publisher, the editors and the reviewers. Any product that may be evaluated in this article, or claim that may be made by its manufacturer, is not guaranteed or endorsed by the publisher.

## Footnotes

^1^Throughout the manuscript, we use identify-first language (“autistic individuals”) rather than person-first language (“individuals with autism”), and use the terms “neurotypical(s)” and “non-autistic(s)” interchangeably, to reflect the preferences of many in the autistic community (Vivanti, 2020; Bottema-Beutel et al., 2021).
